# Red Blood Cell Homeostasis: Pharmacological Interventions to Explore Biochemical, Morphological and Mechanical Properties

**DOI:** 10.3389/fmolb.2016.00010

**Published:** 2016-03-29

**Authors:** Judith C. A. Cluitmans, Federica Gevi, Angela Siciliano, Alessandro Matte, Joames K. F. Leal, Lucia De Franceschi, Lello Zolla, Roland Brock, Merel J. W. Adjobo-Hermans, Giel J. G. C. M. Bosman

**Affiliations:** ^1^Department of Biochemistry, Radboud University Medical CenterNijmegen, Netherlands; ^2^Department of Ecological and Biological Sciences, University of TusciaViterbo, Italy; ^3^Section of Internal Medicine, Department of Medicine, University of VeronaVerona, Italy

**Keywords:** red blood cell, deformability, morphology, phosphorylation, metabolomics

## Abstract

During their passage through the circulation, red blood cells (RBCs) encounter severe physiological conditions consisting of mechanical stress, oxidative damage and fast changes in ionic and osmotic conditions. In order to survive for 120 days, RBCs adapt to their surroundings by subtle regulation of membrane organization and metabolism. RBC homeostasis depends on interactions between the integral membrane protein band 3 with other membrane and cytoskeletal proteins, and with key enzymes of various metabolic pathways. These interactions are regulated by the binding of deoxyhemoglobin to band 3, and by a signaling network revolving around Lyn kinase and Src family kinase-mediated phosphorylation of band 3. Here we show that manipulation of the interaction between the lipid bilayer and the cytoskeleton, using various pharmacological agents that interfere with protein-protein interactions and membrane lipid organization, has various effects on: (1) morphology, as shown by high resolution microscopy and quantitative image analysis; (2) organization of membrane proteins, as indicated by immunofluorescence confocal microscopy and quantitative as well as qualitative analysis of vesicle generation; (3) membrane lipid organization, as indicated by flow cytometric analysis of phosphatidylserine exposure; (4) deformability, as assessed in capillary-mimicking circumstances using a microfluidics system; (5) deformability as determined using a spleen-mimicking device; (6) metabolic activity as indicated by metabolomics. Our data show that there is a complex relationship between red cell morphology, membrane organization and deformability. Also, our data show that red blood cells have a relatively high resistance to disturbance of membrane organization *in vitro*, which may reflect their capacity to withstand mechanical, oxidative and osmotic stress *in vivo*.

## Introduction

Despite constant exposure to oxidative and mechanical stress and fast changes in ionic and osmotic conditions, erythrocytes survive for approximately 120 days in the circulation. In healthy individuals, physiological wear and tear leads to the appearance of immune recognition and removal signals, resulting in phagocytosis. This physiological aging process is disturbed by mutations in hemoglobin, membrane proteins or metabolic enzymes (Bosman, 2004; Da Costa et al., 2013; Koralkova et al., 2014), and by various multifactorial, systemic pathological conditions such as inflammation (Dinkla et al., 2012).

RBC homeostasis is regulated by interactions between proteins in and associated with the plasma membrane. These interactions revolve around band 3. As the main component of the two complexes that link the cytoskeleton to the lipid bilayer (the “ankyrin complex” and the “junctional complex”), band 3 plays a central role in the control of cell morphology and deformability. Also, as a binding partner of both deoxyhemoglobin and key enzymes of the glycolysis, band 3 is the link between the oxygen-dependent, metabolic tradeoff between the use of glucose either in glycolysis for production of ATP or in the pentose phosphate pathway for protection against oxidation by production of NADPH (Dzik, 2011). A signaling network revolving around Lyn and Syk kinase-mediated phosphorylation of band 3 is involved in the regulation of many, if not all these processes. Activation of Syk is the initial signal of this pathway and leads to tyrosine phosphorylation of band 3. Consecutively, Lyn is activated and translocates to the membrane where it phosphorylates its target residues on band 3 (Campanella et al., 2005; Ferru et al., 2011).

Mutations in the main RBC proteins are associated with altered morphology, decreased stability, and/or increased removal, many of which result in anemia (Mohandas and Gallagher, 2008). It has been proposed that mutations in membrane proteins that affect horizontal or lateral interactions, such as the spectrin dimer-dimer structure and the spectrin-actin-protein 4.1R junctional complex, lead to elliptocytosis and membrane fragmentation. Mutations in proteins involved in vertical interactions, such as the binding of the spectrin cytoskeleton to the band 3-ankyrin complex, are thought to induce vesiculation-associated membrane loss, and the concomitant appearance of spherocytes (Tse and Lux, 1999; Mohandas and Gallagher, 2008). However, there is no clear association, not even for a protein as central in membrane organization as band 3, between genotype, RBC morphology and clinical phenotype. Also, there is only a limited number of data on the effect of a disturbance of membrane protein interactions on RBC metabolism. Thus, there is an unmet need for a molecular framework that explains the effect of alterations in one protein on the interaction with its partners and the concomitant effects on RBC morphology and function. Such a framework would be of great benefit for understanding the causes of anemia, including the role of vesiculation and the spleen (Willekens et al., 2003).

It was the aim of this study to improve our understanding of the structure-function relationship of the RBC membrane morphology by treating RBCs with agents that disturb the organization of the RBC cytoskeleton-membrane complex through their effect on band 3. DIDS is an inhibitor of band 3-catalyzed anion exchange that, upon binding to its membrane domain, reduces the linkage between the lipid bilayer and the cytoskeleton, resulting in an echinocyte cell shape (Van Dort et al., 2001). Treatment of RBCs with the thiol-alkylating reagent N-ethylmaleimide (NEM) modifies thiol groups in the N-terminal cytoplasmic domain of band 3, and thereby disturbs the ankyrin-band 3 interaction (Haratake et al., 2009; Blanc et al., 2010). NEM also affects the phosphorylation status of band 3, by acting as a protein phosphatase inhibitor (Bordin, 2002). Finally, phosphorylation of band 3 was manipulated by treating RBCs with the phosphatase inhibitor orthovanadate and the Src family kinase-inhibitor PP2, both affecting the association of band 3 with the cytoskeleton (Low et al., 1987; Brunati et al., 1996, 2000; Pantaleo et al., 2009; Ferru et al., 2011).

We compared the morphological, structural and functional effects of these treatments. Even for interventions that all affect the band 3 complex, a diversity of structural and functional changes was observed. Our data show that the main current theories, in which changes in cell morphology are directly linked to disturbances in horizontal linkage within the lipid bilayer, or to vertical linkages between the lipid bilayer and the cytoskeleton, do not have sufficient heuristic power to explain all the structural and functional changes.

## Materials and methods

### Sample collection and ethical issues

Fresh RBCs were isolated from five ml whole blood (EDTA) from healthy volunteers. RBCs were washed using Ringer solution (125 mmol/L NaCl, 5 mmol/L KCl, 1 mmol/L MgSO_4_, 2.5 mmol/L CaCl_2_, 5 mmol/L glucose, 32 mmol/L HEPES/NaOH, pH7.4) by repeated centrifugation (5 min, 1550 × g). This study was carried out in accordance with the CCMO guidelines of the Medical Ethical Committee of the Radboud University Medical Center and with written informed consent from all subjects. All subjects gave written informed consent in accordance with the Declaration of Helsinki.

### Treatments of the RBCs

RBCs (2% hematocrit) were incubated for 30 min at 37°C in Ringer solution containing 100 μM 4,4′-diisothiocyanostilbene-2,2′-disulfonic acid (DIDS, Sigma-Aldrich, St. Louis MO, USA) or 50 μM N-ethylmaleimide (NEM, Sigma-Aldrich, St. Louis MO, USA). Phosphorylation was manipulated by incubation with 100 μM orthovanadate (Sigma-Aldrich, St. Louis MO, USA) or 20 μM PP2 (Sigma-Aldrich, St. Louis MO, USA). PP2 was dissolved in DMSO and then diluted in Ringer to a solution with a final concentration of 0.1% DMSO. After treatment, the RBCs were washed twice in Ringer solution by centrifugation (3 min, 400 × g) before analysis.

### Cell classification

Cells were resuspended in Ringer solution, poured into Lab-Tek chambered coverglass (Thermo Fisher Scientific, Rochester NY, USA) and random field pictures were taken for morphological classification. Microscopy was performed on a TCS SP5 confocal laser scanning microscope (Leica Microsystems, Mannheim, Germany) equipped with an HCX Plan-Apochromat 63X/N.A. 1.2 water immersion lens. Image J version 1.45j (U. S. National Institutes of Health, Bethesda MD, USA, http://imagej.nih.gov/ij/) was used for image analyses.

### Immunofluorescence

After treatment, the RBCs were fixated and stained as described before (Matte et al., 2010). 1% BSA was used instead of fish skin gelatin for blocking purposes. Primary antibodies and relative dilutions were: mouse anti-stomatin (GARP-50, kindly provided by Rainer Prohaska, University of Vienna, Austria) at 1:200, mouse anti-band 3 N-terminal domain (B3-136, Sigma-Aldrich, St. Louis MO, USA) at 1:100 and mouse anti-band 3 C-terminal domain (IVF12, Developmental Studies Hybridoma Bank, Iowa City, IA, USA) at 1:100. The secondary antibody was goat anti-mouse Alexa-488 (Invitrogen, Carlsbad CA, USA) at 1:1000 dilution. Image analysis and single cell fluorescence determination were carried out using ImageJ version 1.45J.

### Phosphatidylserine (PS) exposure

After treatment, two million RBCs were resuspended in 25 μL of Ringer and incubated for 45 minutes at room temperature in the dark with Fluos–labeled annexin V (1:25, Roche, Indianapolis, IN) to label exposed PS. After washing, the RBCs were analyzed with a flow cytometer (FACSCalibur, BD Biosciences, Franklin Lakes NJ, USA) and Cellquest Pro software version 6.0 (BD Biosciences, Franklin Lakes NJ, USA) was used. Data analysis was performed with Cyan Summit software v4.3 (Beckman-Coulter, Fullerton CA, USA). Results are expressed as percentages of annexin V–positive RBCs.

### Measurement of acetylcholinesterase (AChE) activity

AChE activity was measured using Ellman's method (Salzer et al., 2002), using 2 × 10^6^ cells or, for vesicle measurements, the supernatant of 2 × 10^7^ cells.

### Microfluidics

RBC deformation within the microcapillaries was simulated with a microfluidic device as described before, using 10 × 7 μm channels (Cluitmans et al., 2014). Cells were diluted to a hematocrit of 2% in Ringer solution with 1% BSA. Perfusion of the cell suspension through the microfluidic channels was done with computer-controlled syringe pumps (neMESYS, Cetoni, Germany) for accurate adjustment of the flow rates. In all experiments a constant volumetric flow rate of 25 μl/h was used, which corresponds to a flow velocity of 0.63 cm/s in the narrow channel. Images of up to 1250 frames per second of the field-of-view were recorded for quantitative image processing. Cell flow was observed through a 100X oil immersion objective (Olympus UPLFLN 100X, N.A. 1.30) using an optical microscope (IX71, Olympus B.V., The Netherlands) equipped with a high speed CMOS camera (Phantom high speed camera, Vision Research, UK). Snapshots of RBC shape were obtained using a short exposure time of 10 ms. A sequence of images of 20,000 in each run was recorded. The RBCs in these images were analyzed using the length and width of a rectangular box enclosing the deformed RBC measured by a macro developed for automated image processing using ImageJ. With these values, the deformation index (DI), defined as the ratio between the sides (DI = length/width) was calculated.

### Bead-sorting device

RBC deformability was assessed using a bead-sorting device that mimics the mechanical deformation that RBCs experience in the spleen (Deplaine et al., 2011). RBCs were treated as described above, after which the RBCs (1 × 10^8^) were labeled with CFSE diacetate (Thermo Fisher Scientific). A 2% hematocrit suspension (600 μl) consisting of 5% treated/labeled and 95% untreated/unlabeled RBCs in Ringer with 1% BSA was passed through the bead-sorting device at a flow rate of 60 ml/h. The untreated/unlabeled RBCs acted as both facilitators of steady perfusion of the cells of interest, and as an internal control for deformability. The percentages of CFSE-positive cells in the various samples were determined by flow cytometry (100,000 cells), and the percentages in the retained and downstream fractions were normalized to the labeled cells in the upstream samples.

### Phospho-immunoblotting

Membrane fractions from control and treated red cells were prepared as previously reported (De Franceschi et al., 1998, 2011; Iolascon et al., 2009). Proteins were separated by 1D-electrophoresis, transferred to membranes, and probed with the following specific antibodies: anti-Lyn antibody (2732, Cell Signaling, USA), anti-phospho-Lyn (P-Lyn; Cell Signaling, USA), anti-Syk (Cell Signaling; USA) and anti-phospho-Syk (P-Syk; Cell Signaling; USA); anti-SH-PTP1 (SantaCruz; USA); Phospho-Tyr 564 SH-PTP1 (anti-P-SH-PTP1 564; Cell Signaling, USA). Densitometric analysis of band intensities was carried out by Quantity One analysis software (Bio-Rad, Hercules, CA, USA) (De Franceschi et al., 1997, 2011; Perrotta et al., 2005; Lupo et al., 2016).

### Metabolomic analysis

Metabolomes were charted as described before (D'Alessandro et al., 2013). In short, packed RBCs were lysed, extracted with chloroform/methanol, and treated with acetonitrile to precipitate proteins. Metabolites in the lysate were separated on an Ultimate 3000 Rapid Resolution HPLC system with a Reprosil C18 column. MS analysis was performed, both at downstream negative and positive ion mode, on an electrospray hybrid quadropole time-of-flight mass spectrometer MicroTOF-Q (Bruker-Daltonik, Germany) equipped with an ESI-ion source. Data were acquired with a stored mass range of *m/z* 50–1200. The SmartFormula3D TM software (Bruker Daltonics) was used to calculate molecular formulae based on the isotopic and fragmentation patterns.

## Results

### Cell morphology and membrane organization

**Figure 1:**
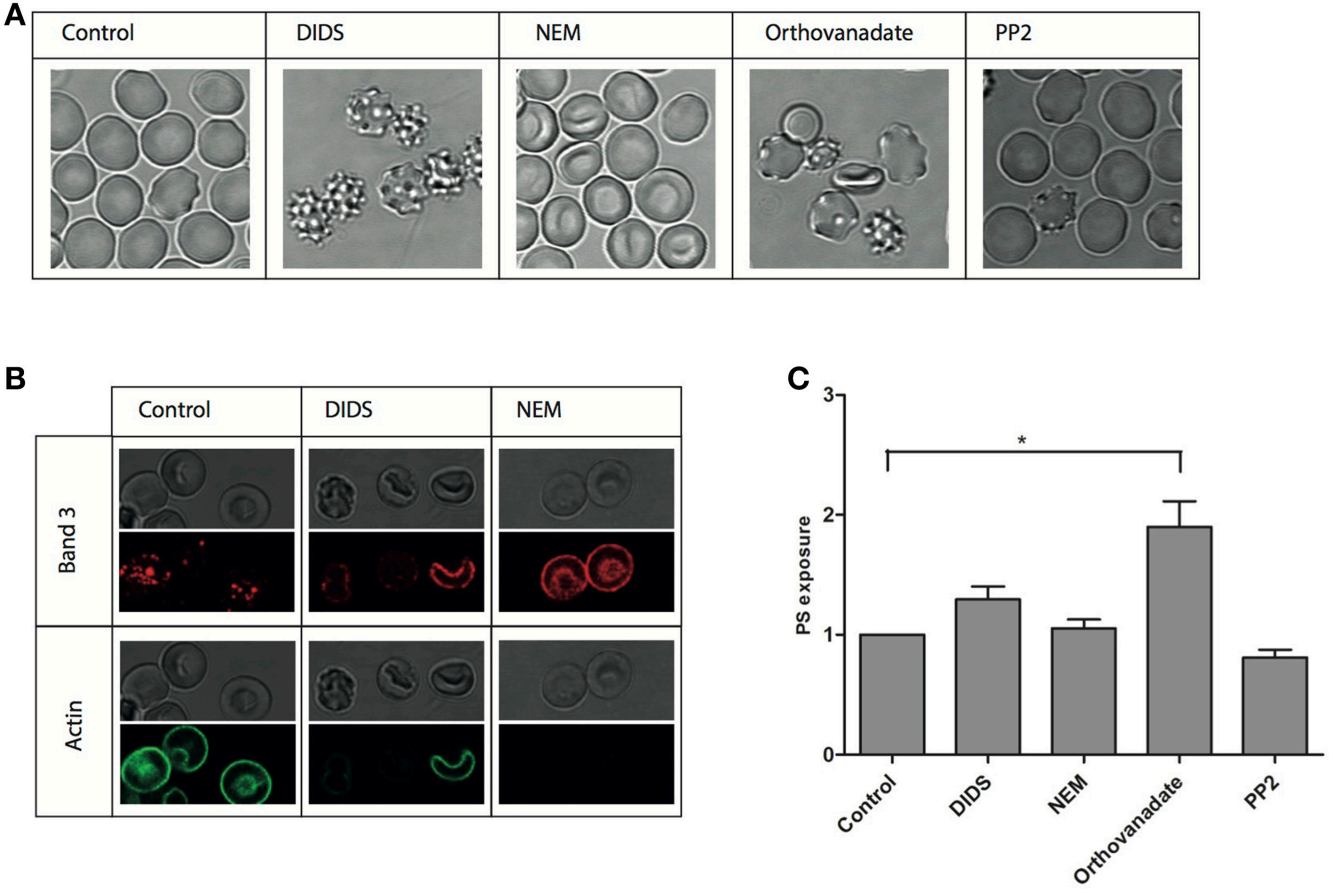
**Morphological and structural changes after treatment of RBCs**. RBCs were treated with various reagents and analyzed as described in Materials and Methods. **(A)** representative RBC morphologies after treatment of fresh RBCs from a healthy volunteer; **(B)** immunofluorescent staining of band 3 and actin; **(C)** PS-positive RBCs expressed relative to the control (*N* = 3, ^*^P < 0.05).

To better define the structure-activity relationship of band 3 and RBC morphology, we subjected RBCs to a series of treatments known to interfere with band 3 function. Remarkably, in spite of this restriction in the choice of compounds, very different effects were observed. Incubation with DIDS at concentrations known to induce band 3 crosslinking as well as the loss of low and high affinity ankyrin-binding sites, induced the formation of stable echinocytes, as described before (Van Dort et al., 2001; Ferru et al., 2011; Mrówczyńska et al., 2012). In contrast, NEM, that affects the conformation of the cytoplasmic domain did not have a distinct effect on morphology (Figure 1A), which is in agreement with previous observations (Blanc et al., 2010). An overall increase in phosphorylation status induced by the phosphatase inhibitor orthovanadate resulted in the appearance of echinocytes as well as spherocytes (Ferru et al., 2011). Inhibition of Src-family kinase activity by PP2 did not show an effect on cell morphology (Figure 1A).

In order to assess the effect of these treatments on the interactions of band 3 with its partners in the membrane-cytoskeleton complex, we investigated concomitant changes in protein and lipid organization using immunofluorescence confocal microscopy. Remarkably, membrane organization was changed at the molecular level also after treatments that did not have a visible effect on cell morphology. Staining with an antibody against the N-terminal domain of band 3 shows a punctuated pattern in control cells, that, upon treatment with NEM, becomes much more diffuse (Figure 1). Treatment with DIDS, which had a profound impact on morphology, induced a similar pattern, at least in some cells. In contrast, the actin antibody stains the control cells, but not the NEM-treated or some of the DIDS-treated RBCs (Figure 1B).

Lipid distribution can also be influenced by changes in the membrane-cytoskeleton complex organization, with phospatidylserine (PS) exposure on the outside of the cell membrane being the most prominent change, and associated with vesiculation and aging (Kuypers and de Jong, 2004). However, only orthovanadate led to an increase in PS exposure, indicating that the disruption of the organization of band 3 complexes had no dramatic effect on PS distribution asymmetry (Figure 1C).

### Phosphorylation

**Figure 2:**
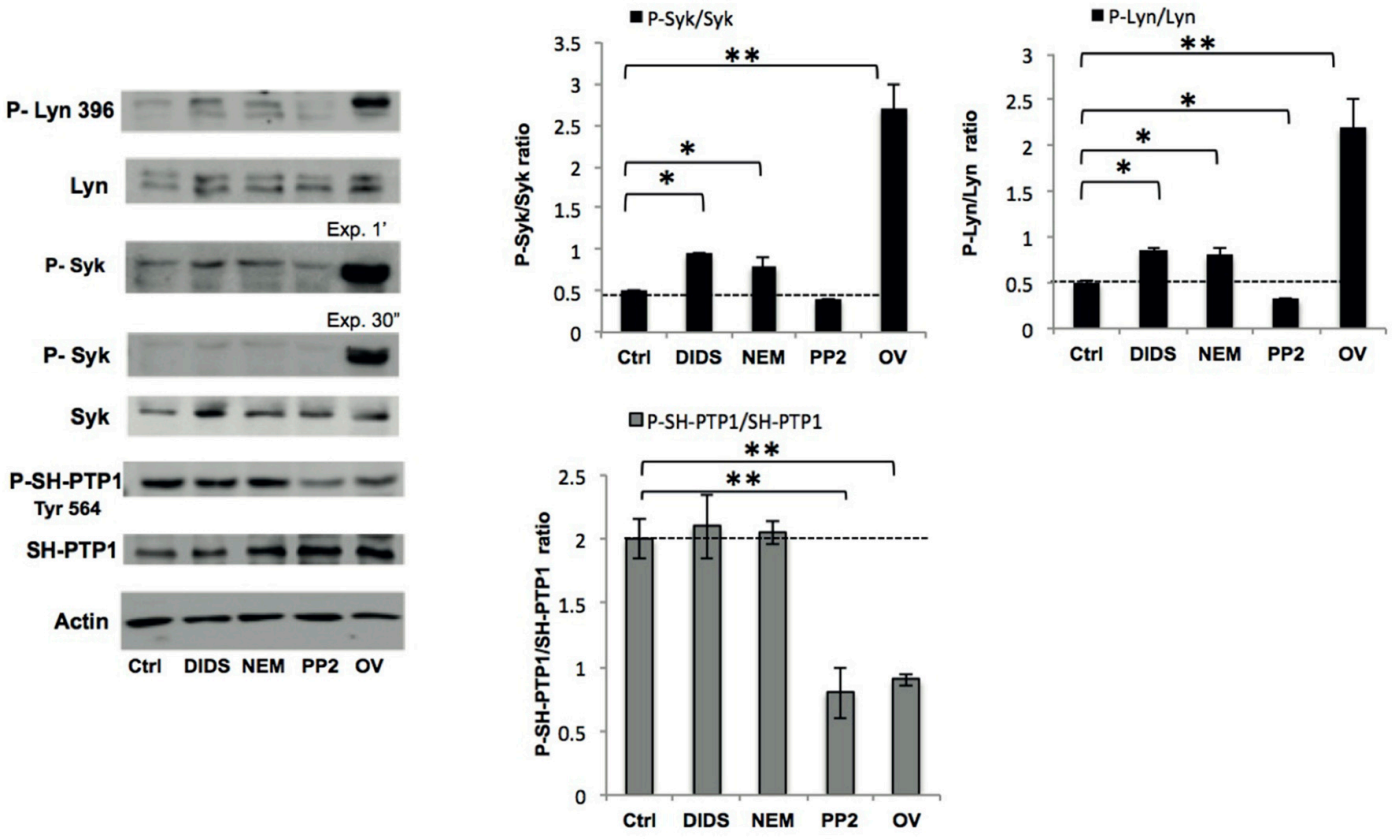
**Phospho-immunoblot analysis of RBC membranes after treatment**. Active Lyn (phospho(P)-Lyn 396), total Lyn, active Syk (phospho (P)-Syk), total Syk, and active SH-PTP1 (phospho(P)-Tyr 564 SH-PTP1) were evaluated using specific antibodies (see also Materials and Methods). The representative experiment shown is one of five similar experiments. Actin was used as loading control. The right panels show the data of a densitometric analysis of the immunoblots; data are shown as means ±SD (*n* = 5; ^*^P < 0.05; ^**^P < 0.02 compared to control RBCs).

We previously showed that the Syk-Lyn pathway plays a key role in the modulation of membrane-cytoskeleton protein-protein and protein-lipid bilayer interactions (Pantaleo et al., 2010; De Franceschi et al., 2011; Lupo et al., 2016). Using cell fractionation and immunoblot analysis, we evaluated the effect of various treatments on membrane translocation of phospho-Syk (P-Syk) and phospho-Lyn (P-Lyn), the active forms of these kinases (De Franceschi et al., 2011). DIDS, NEM and orthovanadate led to Syk and Lyn activation (Figure 2). In PP2-treated cells we found a significant reduction in the membrane association of P-Lyn compared to control cells, whereas no differences in membrane association of P-Syk were observed (Figure 2). This finding is in agreement with previous reports (Pantaleo et al., 2009; De Franceschi et al., 2011). We then evaluated the phosphorylation status of SH-protein tyrosine phosphatase-1 (SH-PTP1), as this enzyme has been functionally linked to Lyn in various cell models (De Franceschi et al., 2008; Nagao et al., 2014). Interestingly, we observed a reduction in SH-PTP1 activation (P-SH-PTP1 Tyr 564) in RBCs treated with the Lyn inhibitor PP2 (Figure 2), suggesting that Lyn may be a downstream regulator of SH-PTP1 in human RBCs.

### Deformability

Deformability plays a key role in RBC functioning *in vivo* and is closely related to the cytoskeleton-membrane interactions. To further complement the morphological studies with a dynamic assessment of deformability, we studied changes in deformation capacity with two techniques that simulate circulation through microcapillaries and passage through the spleen *in vivo*.

**Figure 3:**
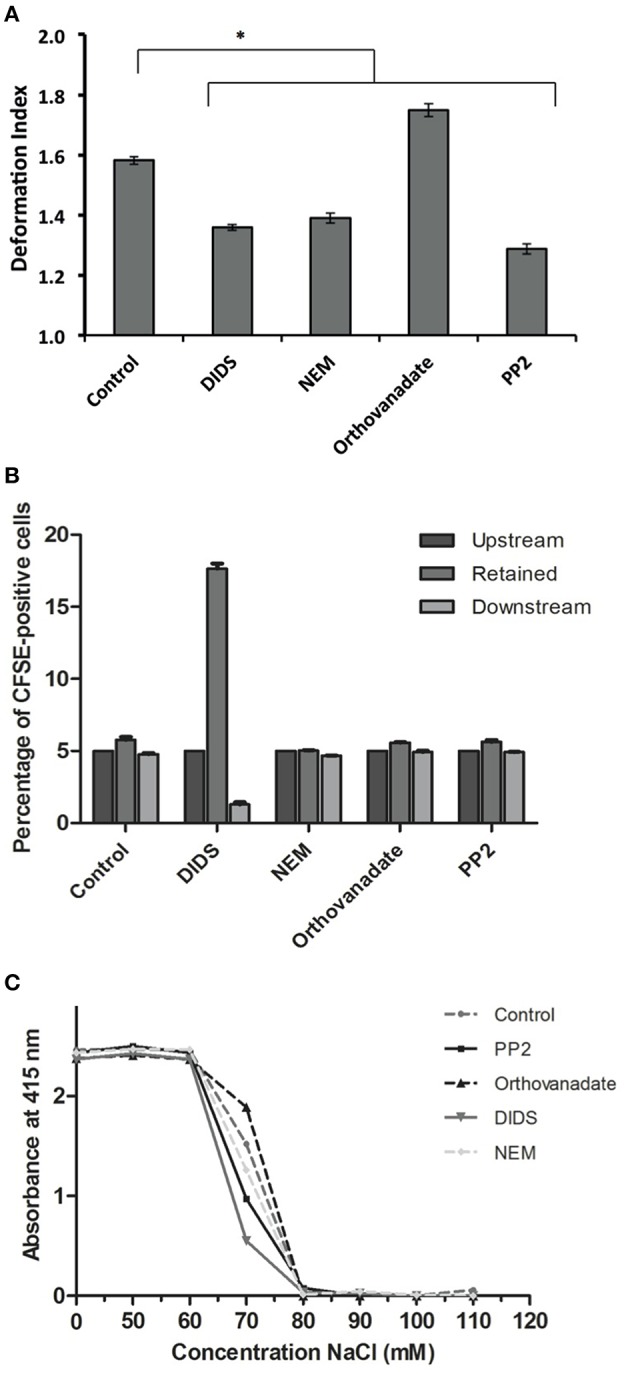
**Deformability characteristics after treatment of RBCs**. RBCs were treated as described in Materials and Methods. **(A)** deformability and relaxation as measured with a microfluidic device; **(B)** deformability and retention as assessed with a spleen- mimicking device; **(C)** an osmotic fragility assay to determine changes in the surface/volume ratio. The values in **(A)** are the mean ± SE of at least 200 cells, ^*^P < 0.05; the values in **(B)** are the mean ± SD of four measurements; the values in **(C)** are the mean of three measurements with a standard deviation of less than 0.02 absorbance units.

Treatment with DIDS and NEM led to a decrease in deformability in the microchannels of a microfluidic device mimicking the microcapillaries (Cluitmans et al., 2014), as did treatment with the Lyn kinase inhibitor PP2 (Figure 3A). In contrast, an increase in the phosphorylation state upon treatment with orthovanadate resulted in an increase in deformability (Figure 3A). When RBCs were passed through a bead-sorting device, mimicking the inter-endothelial slits in the spleen, the capacity to deform was significantly impaired after treatment with DIDS, whereas the other treatments had no effect (Figure 3B). On the other hand, incubation with DIDS was the only treatment that did not impair the capacity of RBCs to elongate under large shear stress in the LORCA ektacytometer or in the ARCA rheoscope (data not shown). These findings are similar to those previously reported for DIDS and NEM (Chasis and Mohandas, 1986; Van Dort et al., 2001; Blanc et al., 2010). Vesiculation may be an important factor that influences deformability by its effects on the surface-to-volume ratio. The latter also affects osmotic fragility. Therefore, in order to assess if changes in deformability where related to changes in surface-to-volume ratio, we determined the osmotic fragility of the treated cells. Almost all treatments led to a decrease in osmotic fragility, with the exception of treatment with orthovanadate, which had the opposite effect (Figure 3C).

### Vesicle formation

**Figure 4:**
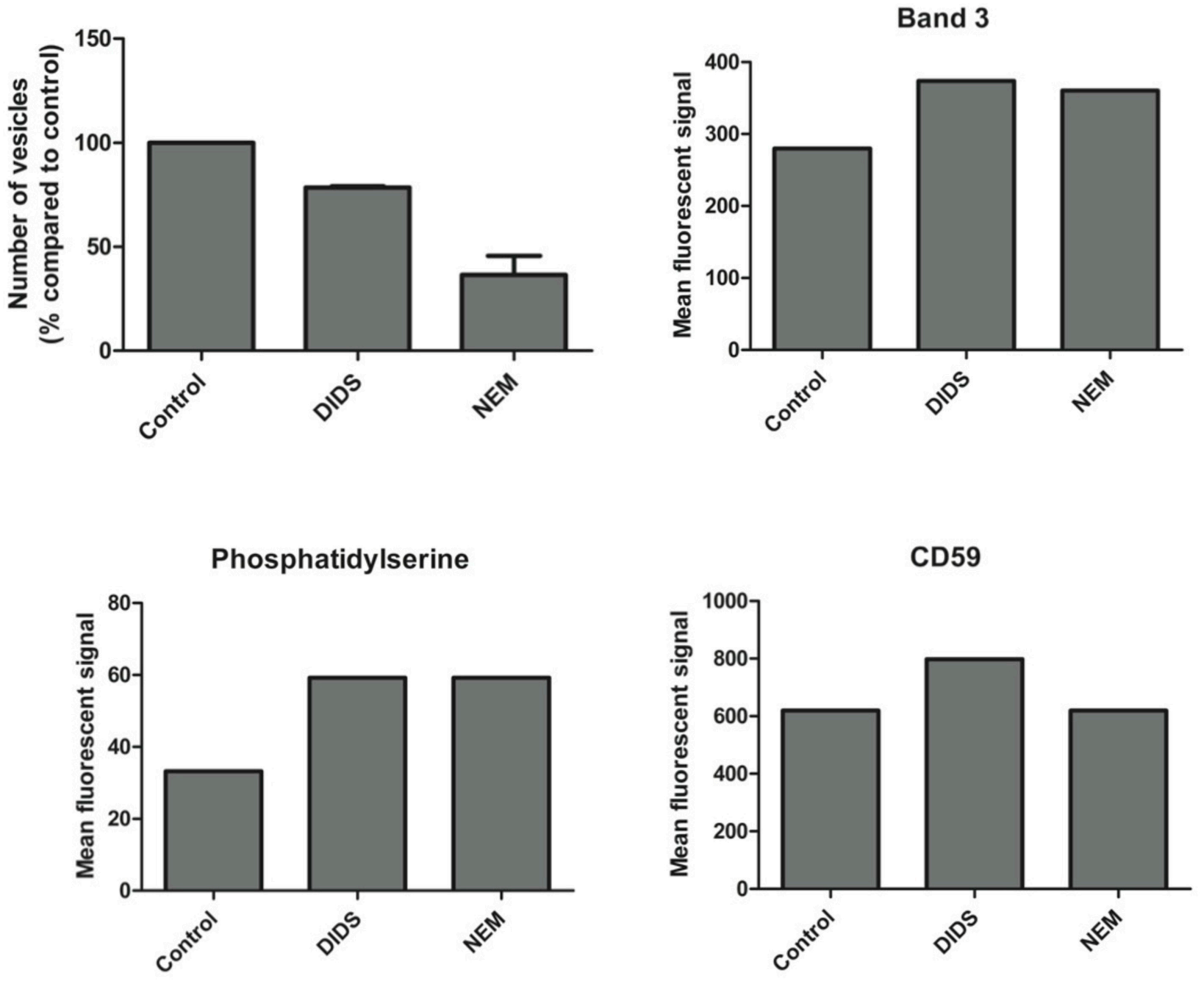
**Vesicle numbers and composition after DIDS and NEM treatment of RBCs**. Cells were treated and the resulting vesicles were quantitated and analyzed by flow cytometry and immunofluorescence as described in Materials and Methods. The data presented are the results of a series of pilot experiments.

It has been postulated that changes in membrane structure that disrupt the binding of the cytoskeleton to the lipid bilayer induce vesicle formation (Sens and Gov, 2006; Mohandas and Gallagher, 2008; Da Costa et al., 2013). Therefore, we measured vesicle number and composition after treatment with DIDS and NEM. All treatments—including the control incubation - induced vesiculation, but the number of vesicles and the composition differed between the control and the treatments. DIDS and NEM treatment both led to a decrease in vesicle production (Figure 4), but the vesicles that were formed did contain more of the integral membrane protein band 3, the lipid-anchored protein CD59, and displayed more of phosphatidylserine (PS) than the control vesicles (Figure 4). These observations support previous conclusions from proteomic analyses that vesicles may originate by different mechanisms, depending on which molecular linkage in the membrane-protein network is broken (Sens and Gov, 2006; Bosman et al., 2010, 2012). To investigate whether treatments led to a breakage of protein contacts with subsequent release of specific proteins in vesicles, we used the GPI-anchored acetylcholinesterase as an independent, quantitative parameter of vesicle production (Salzer et al., 2002). In line with our results for band 3 and CD59, there was a treatment-associated effect. RBC acetylcholinesterase activity was decreased 20% for DIDS, 10% for NEM and 5% for orthovanadate, with a concomitant increase in the acetylcholinesterase activity in the vesicle fractions.

### Metabolism

**Figure 5:**
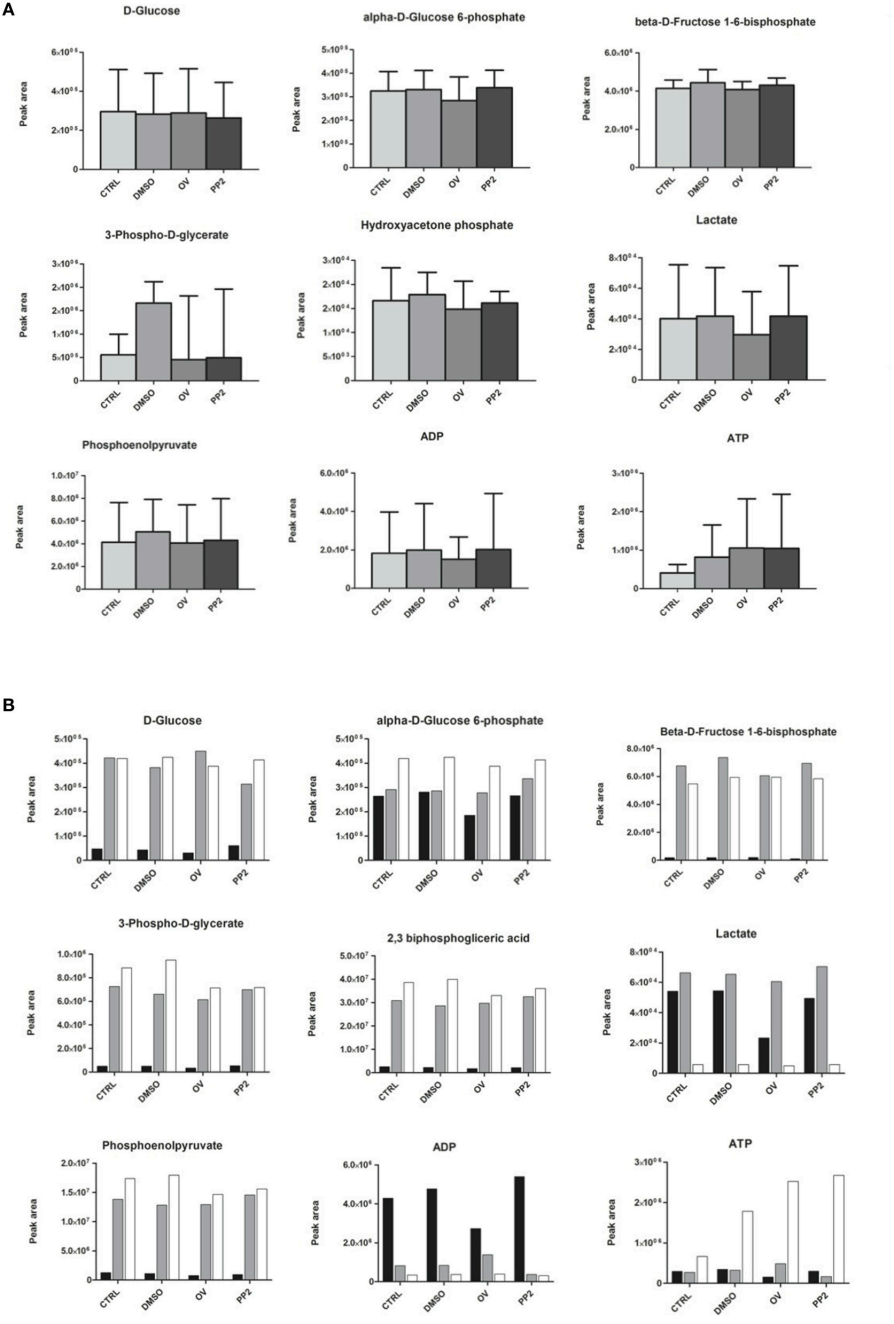
**The effect of manipulation of phosphorylation on red blood cell metabolism**. **(A)** glycolysis intermediates, presented as the means of three donors with standard deviations; **(B)** glycolysis intermediates of three individual donors. For details see Materials and Methods.

Next to having an impact on RBC morphology and dynamic behavior, changes in band 3 conformation and phosphorylation may affect glycolysis as well as the pentose phosphate pathway (PPP; Campanella et al., 2005; Dzik, 2011; Ferru et al., 2011). In a series of pilot experiments, we found that incubation of RBCs with DIDS had an inhibitory effect on glycolysis (Cluitmans et al., unpublished). In physiological circumstances, i.e., at ample glucose supply, a decrease in the rate of the glycolysis is accompanied by an increase in the activity of the pentose phosphate pathway (Dzik, 2011). In line with this mutual interdependence, the same preliminary data indicated that incubation with DIDS resulted in an increase in NADPH, the primary product of the pentose phosphate pathway. In order to explore the underlying, physiological signaling mechanisms, we examined the effect of manipulation of phosphorylation signaling on the metabolomic profile, using the RBCs of three individuals. In contrast to our findings for DIDS, our data did not show significant effects of manipulation of phosphorylation on the concentration of glycolysis metabolites (Figure 5). Orthovanadate increased the concentration of cyclic AMP, indicating an overall effect on intracellular signaling, but had no effect on inositol-1-phosphate and phosphatidylinositol-3,4,5,-trisphosphate levels (Cluitmans et al., data not shown). Also, we found no consistent effects of orthovanadate or PP2 on the pentose phosphate pathway intermediates, nor on the reduced gluthation (GSH)/oxidized gluthation (GSSG) ratios. Incubation with PP2 did not result in any significant changes in the concentrations of the glycolysis or pentose phoshate pathway intermediates (Figure 5). However, our data show considerable inter-individual variability in the susceptibility to such manipulations. This susceptibility extends to the effects of DMSO, as a solvent control for PP2 (Figure 5).

## Discussion

Various mechanisms have been postulated for the generation of abnormally shaped RBCs. These theories revolve around a weakening of the linkage between the cytoskeleton and the lipid bilayer. After an initial screening of a number of compounds, we analyzed the effects of the band 3-directed agents DIDS, NEM, PP2 and orthovanadate on various morphological and functional RBC characteristics, protein localization at the plasma membrane and RBC-derived vesicles. Overall, our data warrant a critical revision of the current paradigms and of the experimental approaches and pharmacological interventions commonly used to investigate them.

### Cell morphology and membrane organization

A comparison of the cell morphology and confocal immunofluorescence data shows that changes in organization of the membrane-cytoskeleton network are not always accompanied by changes in cell morphology, and *vice versa* (Figure 1). Immunoblot analyses of band 3 and spectrin did not reveal any clear concomitant differences in membrane protein composition (data not shown), but did show differences in membrane protein phosphorylation state (Figure 2). The effects of DIDS and NEM on phosphorylation are remarkable, as both treatments do not directly interfere with kinase activity. The absence of a relation between structural changes at the membrane protein level and RBC morphology has been reported before, most notably in the case of aging-associated changes in band 3 conformation (Willekens et al., 2008).

Measurement of PS exposure indicated that only treatment with orthovanadate resulted in a significant disturbance of PS distribution (Figure 1C). This may be due to an increase in phosphorylation status of integral membrane proteins, but we cannot exclude the possibility that orthovanadate inhibits the activity of calcium-extruding ATP-ases with a concomitant rise in intracellular calcium concentration, resulting in increased PS exposure (Smriti et al., 2007; Bogdanova et al., 2013). Also, although in erythrocytes band 3 is the most likely target of DIDS, it cannot be excluded that some effects of DIDS may be due to interference with other anion transporters, such as the voltage-dependent anion channel (Sridharan et al., 2012). Again, a comparison of PS exposure with cell morphology (Figure 1A), showing the same echinocytic effect of both DIDS and orthovanadate, indicates the absence of a consistent correlation between cell morphology and membrane organization.

It has been stated that changes in cell morphology are mainly caused by surface area loss, due to loss of vertical linkage between the lipid bilayer and the cytoskeleton and/or to loss of lateral protein interactions (Da Costa et al., 2013). The results of our vesicle analyses suggest that changes in cell morphology may not always be due to loss of membrane *per se*. A combination of quantitative and qualitative vesicle analyses strongly indicates that proteins are lost from the cell membrane as a result of breaking contacts in the membrane-cytoskeleton complex. Each disturbance of membrane organization results in a specific vesicle population, and an associated loss of specific proteins. A similar conclusion, that was based on theoretical considerations, has been substantiated by a comparison of the vesicles generated during aging *in vivo* with those generated during aging *in vitro* in blood bank storage conditions (Sens and Gov, 2006; Gov et al., 2009; Bosman et al., 2010, 2012). In addition, comparison of the vesicle data with the PS data show that changes in membrane lipid organization may be associated with, but are not essential for, vesicle generation.

### Cell morphology, membrane organization and deformability

There is a notable lack of correlation between morphology and deformability in the capillary-mimicking circumstances of the microfluidic device. For example, an echinocytic morphology induced by DIDS is associated with a decrease in deformability, whereas the deformability of the morphologically indistinguishable echinocytes induced by orthovanadate is increased (Figures 1A, 3). It should also be noted that, although DIDS and orthovanadate did not induce a shape change in all the cells, deformability was changed for the whole cell population. Furthermore, after treatment, misshapen cells behaved the same in the microfluidic device as cells with a normal morphology, suggesting that the effect on deformability is the direct result of changes in membrane organization and that shape is not a predictor of dynamic RBC behavior. In the data we have obtained so far, no consistent correlation can be found for changes in morphology, deformability, or PS exposure (Cluitmans et al., 2012), while the latter parameters are expected to correlate with retention in the spleen. The same conclusion was drawn before for the RBCs of patients with neuroacanthocytosis. Acanthocytes did not show any differences in deformability compared to control cells (Cluitmans et al., 2015).

Conclusions on the effect of a change in membrane organization on deformability are likely to depend on the degree and type of mechanical stress employed. All treatments, except for DIDS, decreased deformability when measured under high shear stress in an ektacytometer (Cluitmans et al., data not shown), while in the spleen-mimicking device only DIDS led to a decrease in deformability. These differences trigger the question on the biological relevance of the commonly used deformability measurement techniques. Since the bead-sorting and the microfluidic device measure deformability under physiological conditions-mimicking circumstances, these techniques seem to be preferable. It remains to be established if and how the method of analysis itself contributes to the observed effects. For example, high mechanical or osmotic stress may themselves induce vesiculation (Ferru et al., 2011), and thereby reduce deformability in susceptible RBCs, that have a normal deformability in less stressful conditions. This could explain the strongly reduced capacity of the DIDS-treated cells to pass the spleen-mimicking device (Figure 1), and normal behavior in an ektacytometer. Echinocytes may be more susceptible to vesicle formation in these circumstances, which would lead to loss of membrane and cell surface and thereby of deformation capacity.

### Cell morphology, membrane organization and metabolomics

Our data show that it is possible to manipulate RBC metabolism by interfering with membrane organization *in vitro*, as has been observed for stomatocytosis and sickle cell disease *in vivo* (Darghouth et al., 2011a,b). Different treatments had different effects on the concentrations of intermediates of the glycolysis, of the closely associated pentose phosphate pathway and on the glutathione concentration, albeit with a strong inter-individual variance. Based on the glycolysis-enhancing effect of deoxygenation, which reduces the binding of band 3 to the cytoskeletal components ankyrin and/or adducin (Dzik, 2011), we postulated that interference with this binding *in vitro* would result in an increase in glycolysis products and a concomitant decrease in the pentose phosphate pathway and in the reduced glutathione concentration. Indeed, in some individuals, the effect of DIDS confirmed the role of band 3 in regulation of the glycolysis, and the concomitant effect on production of ATP and on the redox status through the ratio reduced/oxidized glutathione. The diverse effects of manipulation of the Tyr-phosphorylation status of band 3 on the metabolome indicate an intricate regulation of the main energy-producing and redox status-regulating complexes of the red blood cell. For example, in some individuals the inhibition of Src family kinase (SFK)-mediated phosphorylation of band 3 was associated with an apparent decrease in the rates of both the glycolysis and the pentose phosphate pathway (Figure 5). Together with the asymmetrical effects of inhibition of SFK-mediated phosphorylation and inhibition of phosphatase activity (Figure 4), our data reveal the existence of a phosphorylation-mediated signaling network for regulation of the metabolism that involves more than the binding of glycolytic enzymes to band 3. It is possible that enzymes of the pentose phosphate pathway also bind to band 3, and that glycolysis enzymes also bind to other membrane or cytoskeleton proteins, such as spectrin (Puchulu-Campanella et al., 2013). The latter associations may be regulated by alternative signaling pathways.

Notably, our data suggest large differences in the susceptibility to the treatments between individuals. This may be due to genetic variability in the characteristics of key enzymes in the various metabolomic pathways and/or membrane transporters, or to the inter-individual variability in the natural history of the red blood cells.

Together, our metabolomic findings may provide another starting point for identifying the pathways that regulate RBC metabolism (Dzik, 2011; van't Erve et al., 2014). Also, our findings stress the putative value of the RBC as a model for studying regulation of energy metabolism, with emphasis on the possibility to study glycolysis as an isolated ATP-producing process *in situ*.

## Conclusions

From our pharmacological intervention-based approach aimed to link cell morphology with deformability, membrane protein modification and membrane lipid organization data, our main conclusions, based on the data summarized in Table 1, are:

1. The main current theories on the linkage between (changes in) red blood cell morphology and disturbances in horizontal or vertical associations in the membrane/cytoskeleton complex, require a re-evaluation, since they do not explain all the structural and functional observations;
2. Healthy RBCs have a large capacity to withstand disturbance of membrane organization before this has functional consequences *in vitro*; this may very well be a consequence of the capacity to function in fast-changing conditions *in vivo*, including mechanical, chemical and osmotic stress;
3. In order to understand the molecular mechanisms underlying RBC-mediated pathology, physiologically relevant techniques such as spleen-mimicking and capillary-mimicking devices, reveal functional changes even if the morphology is unaffected. Especially our microfluidic model may reveal the impact of physiological, mechano-sensitive factors on signaling, vesiculation, and metabolism;
4. Detailed analysis of the composition and organization of RBC-derived vesicles is likely to yield more specific and sensitive information;
5. It is possible to manipulate RBC metabolism by manipulating membrane organization. Metabolomic analysis may turn out to be an efficient approach for the identification of the signaling routes that regulate the interaction between the RBC and its organism-wide environment, both in healthy and in pathological circumstances.

**Table 1:** **A summary of the effects of various treatments on morphological and functional characteristics of red blood cells**.

**Treatment**	**Shape**	**Band 3**	**PS**	**Syk/Lyn**	**Deformability**		**Vesicles**	**Glycolysis**
					**MF**	**SMD**		
Control	D	Punctuated	0	0	0	0	0	0
DIDS	E	Diffuse	0	+	−	−	−	−
NEM	D	Diffuse	0	+	−	0	−	0
OV	Sf	ND	+	+	+	0	ND	0
PP2	D	ND	0	−	−	0	ND	0

*Cells were treated with DIDS, N-ethylmaleimide (NEM), orthovanadate (OV) or PP2, followed by morphological and functional analysis as described in Materials and Methods*.

*E, echinocytes; D, discocytes; Sf, sferocytes; PS, phosphatidylserine exposure; MF, microfluidics; SMD, spleen-mimicking device; 0, no change compared with control; +, increased; −, decreased; ND, not determined. For details see Results*.

## Author contributions

JC, GB, RB, and MA conceived the study. JC, FG, AS, AM, and JF performed the experiments and analyzed the data. JC and GB wrote the paper and all other authors edited the manuscript. All authors approved the final version of the manuscript.

## Funding

This study was supported by funding from the E-Rare Joint Translational Call 2009 (European Multidisciplinary Initiative on Neuroacanthocytosis; EMINA) awarded to GB and the E-Rare-2 Call 2012 (EMINA-2) awarded to MA.

### Conflict of interest statement

The authors declare that the research was conducted in the absence of any commercial or financial relationships that could be construed as a potential conflict of interest.
